# The effect of mindfulness combined with exercise compared with a self-management guide on measures of nervous system sensitivity in individuals with chronic pain: a pilot randomised control trial

**DOI:** 10.1007/s11845-025-03947-y

**Published:** 2025-04-15

**Authors:** Orla Deegan, Brona M. Fullen, Catherine M. Doody

**Affiliations:** 1 Department of Rehabilitation Sciences, College of Health Sciences, QU Health, Qatar University, Doha, Qatar; 2 School of Public Health, Physiotherapy and Sports Science, University College Dublin, Dublin 4, Ireland

**Keywords:** Chronic pain, Conditioned pain modulation, Pressure pain thresholds, Quantitative sensory testing, Temporal summation

## Abstract

**Background:**

This study described a sensory profile of participants with chronic pain (CP) in a previously reported feasibility RCT, in terms of quantitative sensory testing (QST) measures and the Central Sensitisation Inventory (CSI).

**Aims:**

The study aimed to explore the changes in QST measures and the CSI in this sample following participation in a mindfulness and physical activity intervention compared to an online self-management guide.

**Methods:**

Participants were randomised into (i) a combined mindfulness and exercise online interactive group or (ii) an online self-management group. Pressure pain thresholds (PPT), temporal summation (TS), conditioned pain modulation (CPM) measures, and the CSI were completed with participants at baseline and post-intervention.

**Results:**

Baseline (*n* = 33) and post-intervention (*n* = 22) measurements were completed. High mean CSI scores (54.69, SD 23.85) were noted at baseline in participants, indicating the presence of central sensitisation [*n* = 33; 70% (*n* = 23) score > 40]. Mean baseline scores for TS were high (2.64, SD 1.60), indicating the presence of pain facilitation, and variable results were observed for baseline PPT and CPM measures. The combined intervention was not found to be superior to a self-management guide in this cohort in terms of changes in PPT, TS, and CPM measures and the CSI.

**Conclusions:**

High baseline CSI and TS scores were identified in the cohort at baseline, with no notable trends identifiable with regard to changes in QST scores or the CSI post-intervention. Further studies are recommended with larger sample sizes in order to understand changes in QST measures following participation in interventions of this nature.

**Supplementary Information:**

The online version contains supplementary material available at 10.1007/s11845-025-03947-y.

## Introduction

Chronic pain (CP) is one of the world’s major health problems and confers a significant burden on economies and healthcare systems [1, 2]. Ongoing sensitisation of the nervous system has been proposed to explain CP conditions [3, 4]. Quantitative sensory testing (QST) is a psychophysical method of testing the perception of touch, vibration, proprioception, pinprick/blunt pressure sensitivity or sensitivity to cold or heat stimuli using stimuli applied under standardised testing protocols, and participants’ self-reported sensory experience is quantified [5]. Using QST, it may be possible to infer the potential underlying mechanisms that may be contributing to the development or maintenance of ongoing pain sensitisation [6, 7]. In the literature, pressure pain threshold (PPT) appears to be the most frequently assessed QST parameter [8]. PPT is a measure of hyperalgesia and defined as the minimum pressure applied at which the subject detects pain [9]. In the context of central sensitisation, reduced PPTs have been reported at testing sites remote from the original pain region [10–12]. Further QST assessments that may be indicative of abnormalities in the central sensory processing of pain include increased temporal summation (TS) [13] and dysfunctional conditioned pain modulation (CPM) [14]. TS is the physiological process of wind-up in which the patient will perceive increasing pain when repeatedly exposed to a painful stimulus of the same intensity. The physiological process is a result of increased firing in spinal secondary neurons due to repetitive C-fibre input and measures the state of hyperactivity in the dorsal horn of the pain facilitation pathways [15, 16]. Central pain mechanisms can also be assessed using CPM to assess the descending pain inhibitory pathways [17]. The CPM paradigm refers to the phenomenon of ‘pain inhibits pain’ where activating pain descending inhibitory pathways with a noxious stimulus (conditioning stimulus) modulates another test stimulus [18]. Information regarding nervous system sensitisation can also be inferred using a patient reported outcome measures (PROM), the Central Sensitisation Inventory (CSI). The CSI is a PROM which has been used to study symptoms of central sensitisation in individuals with CP [19].

In a previously reported feasibility RCT—the ‘MOVE-Online trial’ [20], a combined programme of mindfulness-based stress reduction (MBSR) and exercise was delivered live, online to one group of adults with CP. A second comparison group within the trial had access to an online, evidence-based CP self-management guide, which was accessed independently. This pilot RCT aimed to explore the between group effects of the two interventions on QST measures and the CSI. In addition, this study aimed to describe the sensory profiles of a subgroup of the participants in the MOVE-Online trial in terms of QST measures (PPT, TS, CPM) and using a PROM (CSI).

## Materials and methods

### Study design

The pilot RCT is a secondary analysis of a sub-group of individuals who participated in a parallel-group, feasibility RCT (NCT04899622).

### Participants

Participants in the feasibility RCT were recruited from both a national patient support organisation/registered charity in Ireland for people living with CP, and a waiting list for a multidisciplinary PMP at a consultant-led pain clinic in Dublin, Ireland. The inclusion criteria for the feasibility RCT [20] specified that eligible participants were adults (aged 18 years and older) who had been diagnosed by a consultant in pain medicine or a general practitioner with any type of CP condition persisting for > 12 weeks’ duration and who reported a pain intensity score of > 3 on a Numerical Rating Scale (NRS). In addition, participants were required to provide informed consent and communicate effectively in the English language. Exclusion criteria included need for further diagnostic evaluation (determined by physician), presence of any contraindications to participation in an exercise programme (severe shortness of breath at rest, angina, uncontrolled diabetes or epilepsy, recent (previous 3 weeks) myocardial infarction, pulmonary embolism, deep vein thrombosis or asthma attack), presence of active cancer, concurrent participation (or in the previous 3 months) with any form of psychological, physiotherapy or supervised exercise in addition to the study intervention, presence of substance misuse (diagnosed by a physician), untreated psychosis, acute depression, suicidality and inability to take part in an online exercise programme [20]. Other standard MBSR potential exclusion criteria (e.g. post-traumatic stress disorder, recent bereavement) were investigated as exclusion criteria on an individual basis [21].

### Intervention

The detailed content of the study interventions and details of the therapists who delivered the MBSR and exercise interventions are described comprehensively in the study protocol [20] and are summarised below. Blinding of the therapists or study participants to group allocation was not possible. Participants were randomised into two groups: the MOVE group or the self-management (SM) group. Both of the intervention groups participating in each 9-week programme had 10–13 participants. This group size was chosen to best deliver the intervention format of the MOVE group, allowing for effective group work while also providing the participants with the necessary therapeutic space [22]. Participants in the MOVE group participated in the MOVE-Online programme, a 9-week live, interactive online programme combining group classes of MBSR and exercise. Prior to commencing the intervention, participants took part in a 2-h orientation session live online. Following the orientation, the participants attended 8 weekly online 3-h classes (2 h of MBSR, 1-h exercise). The programme was based on the standard MBSR programme developed at The Centre for Mindfulness in Medicine, University of Massachusetts [21]. In addition, the exercise class, which consisted of a range of general flexibility and strengthening exercises was delivered live online and was advanced every 2 weeks over the programme. Participants randomised to the SM group had access to an online CP self-management guide via the study website (www.move-online.me) which consisted of evidence-based self-help information for people with CP, delivered in biweekly modules. The participants received an email from the lead researcher which included a link to the online material bi-weekly. Participants were free to access the information independently on the study website [21].

### Demographic data

Demographic data was collected for all participants and included age, gender, education level, relationship status, and employment status. Information in relation to participants CP condition and duration of symptoms were also collected.

### Primary outcome measures

The outcomes of interest under examination in this secondary analysis of a subgroup of participants who took part in the larger feasibility RCT are related to measures of nervous system sensitivity. The measures consisted of one PROM, the CSI, and a number of QST measures: PPT, TS, CPM. A subset of participants who were randomised in the feasibility RCT were offered an opportunity to take part in an optional QST testing prior to and following the study interventions.

#### Central sensitisation inventory (CSI)

Participants completed the CSI prior to commencing and following completion of the intervention. The CSI assesses the presence of hypersensitivity by evaluating 25 symptoms that individuals with CP may experience on a 5-point Likert-scale (0 indicates that this symptom never occurs, and 5 indicates that this symptom always occurs) [23]. Good sensitivity (81%) and specificity (79%) values were found for a cut-off score of 40 (out of 100) to identify patients with symptoms of central sensitisation [23].

#### QST testing

Participants consenting to participate in the additional QST measures underwent the testing procedure outlined below prior to commencing the intervention to which they had been assigned and upon completion of the intervention. QST was performed by one investigator to ensure consistency and reproducibility of the results. All testing was performed in the same laboratory testing room with the same equipment. Participants were asked to follow the same medication routine on both testing days. The testing was carried out in the following order: (1) TS remote, (2) TS local, (3) PPT local, (4) PPT remote, (5) CPM.

#### Mechanical temporal summation (TS)

TS was assessed using repeated stimulation by using a weighted pinprick (MRC Systems 256 mN), a method that has been recommended by the German Research Network on Neuropathic Pain [24]. Participants assigned a pain rating, using a 0–10 (NRS), to a single stimulus and 10 s after the single stimulus, a series of ten stimuli using the same weighted pinprick device were applied with a frequency of 1 s^−1^ within the same skin area of about 1 cm^2^. An overall pain rating for the series of 10 pinprick stimuli of the same intensity was given by the participant. This procedure was applied twice on a marked area at the site of pain and at a remote site. To obtain a value for TS, the mean pain rating of the 10 pinprick stimuli was calculated minus the mean pain rating of the single stimulus. TS was assessed locally over the painful area and at a remote site. For participants presenting with upper quadrant pain, the remote site was over the contralateral tibialis anterior muscle, 5 cm distal to the tibial tuberosity. For participants with lower quadrant pain, the remote site was on the contralateral forearm, 5 cm distal to lateral epicondyle of humerus on the volar aspect. In the case of central pain, the remote site was on the right side.

#### Pressure pain thresholds (PPTs)

Pressure was applied using an electronic digital algometer with a probe size of 1 cm^2^ (SomedicAB, Sweden) at an application rate of 30 kPa/s. Participants were instructed to press an automatic cut off button when the first sensation of pressure pain was perceived. Three PPT measurements were taken at two test sites (local to the pain area and at a remote site). The mean of the three measurements was used for analysis [23]. Remote sites were chosen as described for TS.

#### Conditioned pain modulation (CPM)

Pressure pain thresholds were applied as the test stimulus while the conditioning stimulus was cold water immersion at 4 °C [25]. For lower quadrant pain, the following steps were followed: (1) with the participant seated comfortably, PPT was initially measured on the remote site (forearm, 5 cm distal to lateral epicondyle of humerus on the volar aspect); (2) participant immersed the contralateral arm to the elbow in a bath of icy water (4 °C monitored by thermometer) for 60 s. Participants unable to tolerate the water bath for the full 60 s rated their pain before withdrawing the arm (at least 5 on NRS); (3) re-testing of PPTs on the remote site (i.e. the forearm opposite to the side that was immersed in icy water) took place immediately after immersion. For upper quadrant pain, the following steps were followed: (1) with the participant seated comfortably, PPT was initially measured on the remote site (tibialis anterior muscle, 5 cm distal to the tibial tuberosity); (2) the contralateral foot was immersed in a bath of icy water (4 °C monitored by thermometer) midway up the shin for 60 s. Participants unable to tolerate the water bath for the full 60 s rated their pain before withdrawing their foot (at least 5 on NRS); (3) re-testing of PPTs on the remote site (i.e. the lower leg opposite to the side that was immersed in icy water) took place immediately after immersion. The differences between mean PPT values pre-cold immersion and post-cold immersion were calculated. A negative value represented a normal or efficient inhibitory CPM response and a positive value indicated an inefficient or facilitatory CPM response [19].

### Secondary outcome measures

A number of PROMs was completed by participants prior to participating and following completion of the interventions in the feasibility RCT.

- Brief Pain Inventory
  Pain intensity and interference were measured using the Brief Pain Inventory (BPI). The BPI was developed to provide a means of measuring pain intensity and the extent to which pain interferes in the lives of pain sufferers [26]. The scale consists of severity and interference subscales that have shown acceptable internal consistency and were found to be reliable and valid for assessing pain intensity and interference in individuals with chronic non-malignant pain [27].
- Pain Disability Index
  General and domain-specific pain-related disability was measured using the Pain Disability Index (PDI) [28]. Several investigations have provided support for the PDI as a reliable and valid measure of pain-related disability [28, 29]. The PDI has been used to assess pain-related disability in a wide range of debilitating pain conditions including chronic musculoskeletal pain [30, 31].
- 36-Item Short Form Survey
  The SF-36 is considered a measure of health-related quality of life designed to measure physical and mental health based on eight health concepts: physical and social functioning, role limitations due to physical and emotional problems, mental health, vitality, bodily pain and general health perception [32]. It has proven to be adequate for health research, for measurement of both patient and general population health status profiles [33].

### Data analysis

As this study was an exploratory secondary analysis, there was no formal sample size calculation. Baseline characteristics, demographic and clinical data for the participants were reported using descriptive statistics. The exploratory analysis was conducted according to an intention-to-treat principle. Analyses of between group differences were carried out for descriptive purposes post-intervention. All participants who were randomised in the study and took part in the baseline QST testing, regardless of their adherence to attendance of the intervention, were included in the analysis. Linear mixed models on the outcome measures over time were fitted to explore within and between group changes in clinical outcomes of both interventions, which intrinsically adjusts for pre-treatment scores. Effect sizes and 95% confidence intervals for between group contrasts were reported. The estimates of efficacy were interpreted in terms of the width of the 95% confidence intervals (CI) for the between-group effect. IBM SPSS Statistics 27 was used to conduct the analyses [34].

### Missing data

Questionnaire data at baseline and follow-up time points were examined for missing items by the primary researcher. If necessary, participants were prompted to complete any missing answers. Completing the questionnaires online minimised missing data in the PROMs at each time point.

### Ethics and dissemination

Ethical approval for the study was sought and approved by the Mater Misericordiae University Hospital Institutional Review Board (1/378/2124) and the University College Dublin Human Research Ethics Committee (LS-20–76-Deegan-Doody). Participants in the programme provided informed consent via an online consent form prior to randomisation. In addition, participants completing the additional QST testing completed a further in person consent form on the day of testing. All data were managed according to General Data Protection Regulation (EU 2016/679). A study code was allocated to each participant, and any personal information about participants is being stored separately from deidentified data. The study was conducted in accordance with the Helsinki Declaration, ensuring the welfare and rights of all its participants.

## Results

A total of 96 participants were randomised into the feasibility RCT: 49 participants in the MOVE Group and 47 participants in the SM Group. Seventy participants were invited to participate in the QST testing at baseline; however, it was not possible for all invited participants to take part due to the travel restrictions in place due to COVID-19 at the time of the study. The QST measurement procedure was carried out on a convenience sample of 33 participants prior to commencement of the intervention [MOVE group (*n* = 18), SM group (*n* = 15)]. Following participation in one of the two trial arms, 22 participants completed the post-intervention QST measurement procedure [MOVE group (*n* = 14), SM group (*n* = 8)]. Reasons for not attending the follow-up appointment were as follows: a family member is unwell (*n* = 1), work-commitments (*n* = 1), participant unwell at time of testing (*n* = 2), unable to organise transport to testing location (*n* = 2) and five participants did not respond to the invite to attend. Of the 22 participants tested post-intervention, two participants [MOVE group (*n* = 1), SM group (*n* = 1)] did not complete 50% of the intervention required to be considered a programme completer [35].

### Baseline participant characteristics

Baseline characteristics and PROMs for the 33 individuals included in the QST testing protocol can be found in Table 1. In addition, the participating individuals’ self-reported diagnoses are presented in Online Resource 1.

**Table 1:** Participant baseline characteristics

	Total cohort (*n* = 33)	MOVE group (*n* = 18)	SM group (*n* = 15)
Female *n* (%)	26 (78.79%)	13 (72.2%)	13 (86.7%)
Male	6 (18.18%)	4 (22.2%)	2 (13.3%)
Prefer not to say	1 (0.03%)	1 (0.06%)	-
Age, years M (SD)	44.9 (11.3)	45.4 (10.2)	44.1 (12.8)
Number of pain areas* n* (%)			
2–5 pain areas	16 (48.5%)	11 (61.1%)	5 (33.3%)
> 5 pain areas	17 (51.5%)	7 (38.9%)	10 (66.7%)
Number of years with pain			
< 5 years	4 (12.1%)	1 (5.6%)	3 (20.1%)
5–10 years	10 (30.3%)	8 (44.5%)	2 (13.4%)
> 10 years	19 (57.6%)	9 (50%)	10 (66.7%)
CSI mean (SD) (0-100)	54.69 (23.85)	51.11 (22.98)	59.6 (24.96)
BPI severity mean (SD) (0–10)	5.94 (1.89)	5.44 (1.69)	6.53 (1.99)
BPI interference mean (SD) (0–10)	5.86 (2.22)	5.48 (1.96)	6.3 (2.49)
PDI mean (SD) (0–60)	39.12 (17.88)	35.83 (18.99)	43.07 (16.18)
SF-36 PCS mean (SD) (0–100)	33.23 (11.51)	36.10 (10.75)	29.79 (11.79)
SF-36 MCS mean (SD) (0-100)	46.42 (18.51)	47.14 (15.97)	45.55 (21.72)
QST measures			
PPT local mean (SD) kPa	251.06 (207.58)	303.78 (237.36)	187.80 (149.05)
PPT remote mean (SD) kPa	285.67 (187.34)	317.83 (178.38)	247.07 (196.60)
TS mean (SD)	2.64 (1.60)	2.11 (1.40)	3.27 (1.63)
CPM score mean (SD) kPa	6.24 (109.04)	7.61 (64.68)	4.60 (148.63)
CPM efficient (% yes)	57%	50%	66.7%

*n* number of participants, *%* percentage of participants, < less than, > greater than, *SD* standard deviation, *CSI* Central Sensitisation Inventory, *BPI* Brief Pain Inventory, *PDI* Pain Disability Index, *SF-36* 36-Item Short Form Survey, *PCS* Physical Component Score, *MCS* Mental Component Score, *QST* quantitative sensory testing, *PPT* pressure pain threshold, *TS* temporal summation, *CPM* conditioned pain modulation, *kPa* kilopascal, *MOVE group* online interactive programme of MBSR and exercise, *SM group* online self-management guide

### Intention to treat analyses: QST measures

The results of the intention to treat analyses of the QST measures (PPT local, PPT remote and TS) are described in detail in Table 2. During the post-intervention period, there were small between-group differences observed in the QST measures both within and between groups.

**Table 2:** Intention to treat analysis for QST measures and central sensitisation inventory

Outcome measure	Group		Baseline	Post-intervention	*p*-value	Effect size*
PPT local	MOVE group	Mean (95% CI)	231.22 (177.18–285.27)	218.97 (155.57–282.36)		
		Δ (95% CI)		12.26 (− 30.63–55.14)	0.558	0.15
	SM group	Mean (95% CI)	161.47 (102.26–220.67)	221.24 (145.60–296.89)		
		Δ (95% CI)		− 59.78 (− 116.28 to − 3.28)	0.039	0.35
	Group difference			72.03 (1.10–142.96)	0.047	0.07
PPT remote	MOVE group	Mean (95% CI)	313.11 (242.91–383.31)	301.19 (228.33–376.06)		
		Δ (95% CI)		10.92 (−33.30–55.14)	0.612	0.32
	SM group	Mean (95% CI)	214.80 (137.90–291.70)	240.01 (153.81–326.22)		
		Δ (95% CI)		− 25.21 (− 83.08–32.65)	0.375	0.28
	Group difference			36.13 (− 36.68–108.94)	0.313	0.13
TS (ratio score)	MOVE group	Mean (95% CI)	2.11 (1.38–2.84)	1.90 (1.05–2.74)		
		Δ (95% CI)		0.22 (− 0.48–0.91)	0.523	0.01
	SM group	Mean (95% CI)	3.27 (2.47–4.07)	2.93 (1.89–3.97)		
		Δ (95% CI)		0.34 (− 0.57–1.24)	0.446	0.18
	Group difference			− 0.12 (− 1.26–1.01)	0.828	0.09
CSI score	MOVE group	Mean (95% CI)	51.11 (39.63–62.6)	49.26 (39.75–58.76)		
		Δ (95% CI)		1.85 (− 4.75–8.46)	0.571	0.09
	SM group	Mean (95% CI)	59.00 (46.42–71.58)	58.85 (48.50–69.2)		
		Δ (95% CI)		0.15 (− 6.99–7.30)	0.965	0.01
	Group difference			1.70 (− 8.03–11.43)	0.723	0.14

*Δ* change from baseline, *CI* confidence interval, *PPT* pressure pain threshold, *TS* temporal summation, *CSI* Central Sensitisation Inventory, *MOVE group* online interactive programme of MBSR and exercise, *SM group* online self-management guide

^*^Cohen’s *D* computed as mean difference relative to pooled standard deviations (baseline standard deviations used in both within and between group calculations)

### PPT local

The within group difference for PPT local measures showed an increase in PPT local scores with a small effect size for the SM group (mean change score = − 59.78, 95% CI = − 116.28 to − 3.28, *d* = 0.35). In contrast, there was a decrease in PPT local scores in the MOVE group (mean change score = 12.26, 95% CI = − 30.63–55.14, *d* = 0.15). Between group mean differences post-intervention for PPT local scores were found in favour of the SM group and also had a small effect size (mean difference = 72.03, 95% CI = 1.10–142.96, *d* = 0.07).

### PPT remote

The within group difference for PPT remote measures showed an increase in PPT remote scores with a small effect size for the SM group (mean difference = − 25.21, 95% CI − 83.08–32.65, *d* = 0.28). In contrast, there was a decrease in PPT remote scores in the MOVE group (mean change score = 10.92, 95% CI = − 33.30–55.14, *d* = 0.32). Post-intervention between group mean difference for PPT remote measures in favour of SM group also had a small effect size (mean difference = 36.13, 95% CI = − 36.68–108.94, *d* = 0.13).

### TS

Both the SM and MOVE group showed a decrease in TS ratio scores with small effect sizes for both groups (SM group, *d* = 0.18; MOVE group, *d* = 0.1). Between group mean difference TS scores were found in favour of the MOVE group and also had a small effect size (mean difference = − 0.12, 95% CI = − 1.26–1.01, *d* = 0.09).

### CPM

The baseline and follow-up scores for CPM in the MOVE group and the SM group can be found in Table 3. At baseline, in the MOVE group, *n* = 9 (50%) individuals demonstrated efficient CPM, and *n* = 9 (50%) individuals had inefficient CPM. In the SM group, *n* = 10 (66%) had efficient CPM, and *n* = 5 (33%) had inefficient CPM. Post-intervention, 22 participants were tested. Overall, *n* = 15 individuals remained in the same category with regard to efficiency of CPM. Four individuals changed from an inefficient CPM to efficient CPM. Of these four, all four attended the MOVE group. Three individuals changed from having an efficient CPM pre-intervention to inefficient CPM when assessed post-intervention. Of these three, one attended the MOVE group, and two attended the SM group.

**Table 3:** CPM efficiency for the MOVE group and the SM group at baseline and following participation in an online PMP

	Baseline		Post-intervention	
	MOVE group	SM group	MOVE group	SM group
CPM efficient, *n* (%)	9 (50%)	10 (66.7%)	12 (66.7%)	3 (20%)
CPM inefficient, *n* (%)	9 (50%)	5 (33.3%)	2 (11.1%)	5 (33.3%)
			(Drop-out *n* = 4 (22.2%)]	[Drop-out *n* = 7 (46.7%)]
Total	18 (100%)	15 (100%)	18 (100%)	15 (100%)

*CPM* conditioned pain modulation, *MOVE group* online interactive programme of MBSR and exercise, *SM group* online self-management guide, *%* percentage of participants

### CSI

The within group difference for CSI showed a decrease in scores with a small effect size for both groups; MOVE group (mean change score = 1.85, 95% CI = − 4.75–8.46, *d* = 0 0.09), SM group (mean change score = 0.15, 95% CI = − 6.99–7.30, *d* = 0.01). Between group mean difference post-intervention for CSI also had a small effect size (mean difference = 1.70, 95% CI = − 8.03–11.43, *d* = 0.14).

### Intention to treat analyses: PROMS

The within and between group differences for the secondary outcome measures—BPI interference, BPI severity, PDI, SF-36 MCS, SF-36 PCS—are described in detail in Online Resource 2. Small non-significant improvements in all scores were found in both groups. Small effect sizes are seen for all between and within group comparisons with the exception of BPI Interference where a moderate effect size is seen in the MOVE group (mean difference 1.24, 95% CI 0.11–2.36, *d* = 0.61).

### Correlations

On the investigation of correlations between the changes in a number of QST measures (PPT local, PPT remote, TS) and changes in a number of PROMs (SF-36 MCS, SF-36 PCS), small correlation coefficients (*r*) were identified. Moderate correlations were identified between changes in PPT local and PDI (*r* = 0.43) and BPI Interference (*r* = 0.34). The correlations are presented in Online Resource 3.

## Discussion

The results of the current study reported that a combined online MBSR and exercise intervention was not found to be superior to an online self-management guide in the analysed cohort of participants with mixed CP conditions in terms of changes in nervous system sensitisation, quantified using QST measures: PPT local, PPT remote, CPM, TS, and the CSI. Furthermore, moderate correlations were found between the changes in PPT local scores and the PDI and BPI Interference PROMs.

This study describes a profile of a group of adults with CP in terms of measures of nervous system sensitisation, prior to participating in a feasibility RCT comparing an online group-based mindfulness and exercise programme to an online self-management guide. In both intervention groups, baseline CSI scores indicated a centrally sensitised cohort with mean scores of > 40 [MOVE group 54.69 (SD 23.85); SM group 51.11 (SD 22.98)] [36]. CSI scores were high when compared to a previous similar RCT [37] investigating individuals with chronic low back pain with mean baseline CSI scores of 40.02 (SD 1.47) and 39.88 (SD 1.47). Furthermore, the mean baseline scores for TS [MOVE group: 2.11 (SD 1.40), SM group: 3.27 (SD 1.63)] were higher than reported in previous studies, demonstrated in the wind-up ratio scores. Georgopoulos et al. [38] investigated cognitive behavioural based physiotherapy treatment in a population of patients (*n* = 97) with chronic low back pain and found median baseline TS scores of 1.0 (95% CI 0.4–2.8). Furthermore, Holm et al. [39] investigated education combined with neuromuscular exercise and strength training in patients with knee osteoarthritis (*n* = 90) and found baseline mean TS scores of 1.9 (SD 1.4) and 2.3 (SD 1.5). CPM scores at baseline showed a high level of variability (MOVE group, 50% individuals efficient CPM; SM group, 66% individuals efficient CPM). In both healthy and CP cohorts, CPM variability has been demonstrated in previous investigations [40]. The wide confidence intervals and variable baseline scores found in both local and remote PPT scores [PPT local: MOVE group 303.78 (SD 237.36), SM group 187.80 (SD 149.05); PPT remote: MOVE group 317.83 (SD 178.38), SM group 247.07 (SD 196.60)] may be due to the range of CP conditions examined in this study (chronic low back pain, fibromyalgia, knee osteoarthritis, etc.), in addition to the small sample size of participants. Notably the current cohort were found to have high baseline pain levels indicated by high mean BPI interference; 5.94 (SD 1.89) and BPI severity; 5.86 (SD 2.22) scores. In addition, 51.5% reported > 5 areas of pain, and 57.6% reported > 10 years of pain. The findings suggest the cohort examined displayed a number of features highly suggestive of central sensitisation; high CSI scores, high TS scores, and a high reported number of pain sites.

In the current study, participants presented with a range of self-reported CP diagnoses (Online Resource 1), with 46% of participants reporting more than one CP diagnosis. Fibromyalgia was the sole diagnosis or part of the CP diagnosis for 30% of participants. Many previous studies investigating changes in measures of nervous system sensitisation have focused on analysing these measures in specific pain conditions: low back pain [41], migraine [42], fibromyalgia [43], knee osteoarthritis [44]. However, it has been shown that in terms of pain processing, variations can vary widely within each diagnosis [45]. For example, in patients with fibromyalgia, some studies have demonstrated lowered inhibitory CPM, suggesting dysfunction in central inhibition when compared to healthy controls; however, healthy controls have also been found to exhibit reduced CPM inhibition [40]. Furthermore, some individuals with fibromyalgia have normal CPM responses while some healthy controls exhibit reduced CPM inhibition [40]. These variations indicate that heterogeneity in pain mechanisms can be substantial between individuals with the same CP diagnosis. Baseline CPM scores varied within the current study cohort with 58% (*n* = 19) of CP participants exhibiting efficient CPM and 42% (*n* = 14) exhibiting an inefficient CPM, with mean CPM scores in both groups demonstrating wide standard deviations.

TS is thought to be related to endogenous excitatory pain mechanisms [46, 47]. Little variability in the wind-up ratio (WUR) measure has been reported in the literature, with most studies consistently reporting WUR values within a narrow range of 1.6–1.7 [48, 49] in both normal and pain states [37, 49]. As previously outlined, the baseline mean WUR scores in the current study were larger than previously reported. Studies have reported that patients with increased TS were considered at risk of developing a pain condition, experienced greater pain severity as pain developed and had a greater tendency to progress from acute to CP [50, 51]. In addition, TS has been found to be higher in fibromyalgia patients compared with healthy controls [40, 52], and as previously noted, 30% of participants in the current study were diagnosed with fibromyalgia (Online Resource 1). Vaegter et al. [41] investigated the effect of different types of exercise on central mechanisms (*n* = 136 participants) in individuals with chronic low back pain and found that isometric exercises, as opposed to aerobic exercise, reduced TS scores. The authors [41] suggested that strengthening exercises might have positive effects on the neuronal hyperexcitability in CP patients that may not be achieved with aerobic exercise. In the current study, the exercise component included a range of strengthening and stretching exercises. Small changes were found in TS ratio scores in both the MOVE group (mean change score = 0.22, 95% CI − 0.48–0.91) and SM group (mean change score = 0.34, 95% CI − 0.57–1.24) with small effects size described for between group differences post-intervention (*d* = 0.09).

In the current study, 70% of the individuals assessed at baseline had scores of > 40 on the CSI. In terms of changes in CSI scores following participation in the intervention, improvements in both groups were seen with small effect sizes (MOVE group: mean change score = 1.85, 95% CI − 4.75–8.47, *d* = 0.09) (SM group: mean change score = 0.15, 95% CI − 267 6.99–7.30, *d* = 0.01). In addition, small between group differences were identified in favour of the MOVE group (*d* = 0.14). A number of previous studies have investigated changes in CSI following participation in an intervention that includes an exercise and pain neuroscience education component. Malfliet and colleagues [37] (*n* = 120) investigated the effect of pain neuroscience education combined with cognition-targeted motor control training on chronic spinal pain. The baseline CSI scores in this study were 40.02 (SD 1.47) in the experimental group (combining pain neuroscience education and cognition-targeted motor control training) and 39.88 (SD 1.47) in the control group (combining education on back and neck pain and general exercise therapy), indicating the presence of central sensitisation at baseline in both groups. Following participation in the programme, the experimental group was found to have reduced CSI scores at 6 months (estimated marginal mean − 5.684; 95% CI − 10.589 to − 0.780) and 12 months (estimated marginal mean − 6.053; 95% CI − 10.781 to − 1.32). The baseline CSI scores indicate centrally sensitised cohorts; however, the baseline CSI scores found in the current study are notably higher and, in addition, have large standard deviations (mean 54.69; SD 23.85) when compared to this study [37]. The high baseline CSI values observed in the current study may further contribute to the small changes seen in this group following participation in the intervention. Furthermore, post-intervention CSI scores were taken immediately post-programme participation in the current study.

The current study was limited by a small sample size as a result of government restrictions on travel due to COVID-19 at the time of testing. Further pre-defined, RCTs with larger sample sizes are recommended to investigate intervention efficacy in terms of changes to nervous system sensitisation. Furthermore, medications, particularly drugs affecting the CNS, may alter the assessment of TS, CPM and PPT [43]; however, discontinuation of medication is also challenging in CP populations. There are ethical concerns when considering the potential pain exacerbation that may be produced because of medication withdrawal and the exclusion of individuals who cannot stop taking medication for testing. In the current study, participants were requested to note medication taken on the day of initial assessment and to follow the same medication regime on the day of their follow-up appointment.

## Conclusion

The current study explores a small sample of participants to investigate how both QST measures and the CSI may provide valuable information regarding the mechanisms underlying the pain experiences of individuals with CP as well as exploring if changes in these measures occur following participation in interventions targeting pain management. Going forward, further studies are required to expand understanding of the role of QST in quantifying changes in nervous system sensitisation following participation in interventions of this nature. Furthermore, studies are recommended with larger samples, using standardised QST testing procedures, to understand the utility of QST testing in CP populations.

## Supplementary Information

Below is the link to the electronic supplementary material.

Supplementary file1 (DOCX 29 KB)

## Data Availability

The data contained in this manuscript is available upon request from the corresponding author.
