# Supplementary material for: The effect of mindfulness combined with exercise compared with a self-management guide on measures of nervous system sensitivity in individuals with chronic pain: a pilot randomised control trial
Source: Ir J Med Sci. 2025 Apr 15;194(3):1167–76. doi: 10.1007/s11845-025-03947-y (PMC12276145; doi:10.1007/s11845-025-03947-y)
Supplement: Supplementary file 1 — Supplementary file1 (DOCX 29 KB) [file 11845_2025_3947_MOESM1_ESM.docx]

**Online Resource 1. Self-reported diagnosis of participants**

| **Diagnosis**  **(participants reporting 1 diagnosis)** | **Number of participants**  **(n)** | **Diagnosis**  **(participants reporting 2 diagnoses)** | **Number of participants**  **(n)** | **Diagnosis**  **(participants reporting >2 diagnoses)** | **Number of participants (n)** |
| --- | --- | --- | --- | --- | --- |
| Low back pain | 6 | Fibromyalgia/ Ankylosing Spondylitis | 1 | Fibromyalgia, Chronic Fatigue Syndrome, Irritable Bowel, Spondylosis | 1 |
| Sciatica | 1 | Fibromyalgia/Bulging discs | 1 | Fibromyalgia/ Arthritis/ Post-surgical pain | 1 |
| Fibromyalgia | 3 | Fibromyalgia/ Osteoarthritis | 1 | Fibromyalgia/ Hypermobility/ Scheuermann’s Disease, Interstitial Cystitis, Lyme disease | 1 |
| CRPS | 1 | Sciatica/ Chronic low back pain | 1 | Fibromyalgia/ Chronic Pain/ Autoimmune disease (unspecified)/ Myofascial Pain Syndrome/ Daily persistent headache chronic migraine syndrome | 1 |
| Neuropathy | 1 | Sciatica/ Migraine | 1 | Neuralgia/ Soft tissue damage/ Complex Pain Syndrome | 1 |
| Osteoarthritis | 1 | Spinal stenosis/Bulging disc | 1 | Fibromyalgia/ Rheumatoid arthritis/ Osteoarthritis/ Enthesitis | 1 |
| Hip bursitis | 1 | Neck pain/ Shoulder pain | 1 | Chronic pelvic pain/ Low back pain/ Prostate floor pain | 1 |
| Post-surgical back pain | 1 |  |  | Back problems-nerve damage / knee pain/ Right arm pain | 1 |
| Post-surgical neck pain | 1 |  |  |  |  |
| Knee osteoarthritis | 1 |  |  |  |  |
| Constant pain | 1 |  |  |  |  |
| **TOTAL** | **18** | **TOTAL** | **7** | **TOTAL** | **8** |

Legend: CRPS, Chronic Regional Pain Syndrome; n, number of participants; %, percentage of participants;

Online Resource 2. Intention to treat analysis for PROMs

| **Outcome Measure** | **Group** |  | **Baseline** | **Post Intervention** | **p-value** | **Effect size*** |
| --- | --- | --- | --- | --- | --- | --- |
| **BPI Interference** | MOVE group | Mean(95% CI) | 5.48(4.42-6.55) | 4.25(3.20-5.29) |  |  |
|  |  | Δ (95% CI) |  | 1.24(0.11-2.36) | 0.032 | 0.61 |
|  | SM group | Mean(95% CI) | 6.3(5.13-7.47) | 5.93(4.81-7.06) |  |  |
|  |  | Δ (95% CI) |  | 0.37(-0.85-1.58) | 0.543 | 0.10 |
|  | Group Difference |  |  | 0.87 (-0.79-2.53) | 0.291 | 0.39 |
|  |  |  |  |  |  |  |
| **BPI severity** | MOVE group | Mean(95% CI) | 5.44(4.56-6.33) | 5.25(4.31-6.20) |  |  |
|  |  | Δ (95% CI) |  | 0.19(-0.55-0.94) | 0.596 | 0.09 |
|  | SM group | Mean(95% CI) | 6.53(5.56-7.50) | 6.28(5.26-7.31) |  |  |
|  |  | Δ (95% CI) |  | 0.25(-0.55-1.05) | 0.525 | 0.16 |
|  | Group Difference |  |  | -0.06(-1.15-1.03) | 0.916 | 0.04 |
|  |  |  |  |  |  |  |
| **Pain Disability Index** | MOVE group | Mean(95% CI) | 35.83(27.29-44.38) | 32.44(23.70-41.19) |  |  |
|  |  | Δ (95% CI) |  | 3.39(-2.46-9.25) | 0.245 | 0.25 |
|  | SM group | Mean(95% CI) | 43.07(33.71-52.43) | 39.42(29.91-48.93) |  |  |
|  |  | Δ (95% CI) |  | 3.65(-2.66-9.95) | 0.245 | 0.16 |
|  | Group Difference |  |  | -0.26(-8.86-8.3) | 0.952 | 0.02 |
|  |  |  |  |  |  |  |
| **SF36 MCS** | MOVE group | Mean(95% CI) | 47.14 (38.11-56.17) | 48.81(38.37-59.26) |  |  |
|  |  | Δ (95% CI) |  | -1.67(-12.30-8.95) | 0.749 | 0.21 |
|  | SM group | Mean(95% CI) | 45.55(35.66-55.44) | 47.50(36.23-58.76) |  |  |
|  |  | Δ (95% CI) |  | -1.95(-13.41-9.51) | 0.730 | 0.09 |
|  | Group Difference |  |  | 0.28(-15.35-15.91) | 0.971 | 0.01 |
|  |  |  |  |  |  |  |
| **SF36 PCS** |  |  |  |  |  |  |
|  | MOVE group | Mean(95% CI) | 36.1 (30.70-41.5) | 38.45(31.34-45.57) |  |  |
|  |  | Δ (95% CI) |  | -2.35(-6.97-2.26) | 0..304 | 0.13 |
|  | SM group | Mean(95% CI) | 29.79(23.87-35.70) | 36.22(28.47-43.96) |  |  |
|  |  | Δ (95% CI) |  | -6.43(-11.38—1.48) | 0.013 | 0.36 |
|  | Group Difference |  |  | 4.07(-2.69-10.84) | 0.277 | 0.39 |

Legend: Δ, change from baseline; CI, Confidence interval; BPI, Brief Pain Inventory; SF36, 36 Item Short Form Survey; MCS, Mental Component Score; PCS, Physical Component Score; MOVE Group, Online interactive programme of MBSR and exercise; SM Group, Online self-management guide.

*Cohen’s D computed as mean difference relative to pooled standard deviations (baseline standard deviations used in both within and between group calculations).

Online Resource 3. Correlations between QST change scores and changes in PROMS (Pearson Correlation R)

| **QST Measure** | **PROM** | | | | |
| --- | --- | --- | --- | --- | --- |
|  | **PDI** | **BPI Interference** | **CSI** | **SF-36 PCS** | **SF-36 MCS** |
| **PPT local (r)** | 0.43 | 0.34 | 0.01 | 0.22 | -0.12 |
| **PPT remote (r)** | -0.07 | 0.22 | -0.21 | 0.19 | 0.08 |
| **TS (r)** | -0.04 | 0.24 | -0.21 | -0.06 | -0.03 |

Legend: PPT, Pressure pain threshold; TS, Temporal summation; PDI, Pain Disability Index; BPI, Brief Pain Inventory; CSI, Central Sensitisation Inventory; SF-36 PCS, Short-Form Questionnaire Physical Component Score; SF-36 MCS, Short-Form Questionnaire Mental Component Score; PROM, Patient Reported Outcome Measure; QST, Quantitative Sensory Testing; r, Pearson Correlation Coefficient.
